# A CASE OF HAEMOPTYSIS DUE TO ENDOBRNCHIAL FIBROMA, A RARE BENIGN TUMOUR OF LUNG

**DOI:** 10.4103/0970-2113.44135

**Published:** 2008

**Authors:** Sibes Kumar Das, Ramendra Sundar Mukherjee, Anirban Das, Amp Kumar Halder, Samirendra Kumar Saha

**Affiliations:** Department of Respiratory Medicine, Calcutta National Medical College & Hospital, 24, Gorachand Road, Kolkata -700014

**Keywords:** Benign tumours of lung, Endobronchial fibroma, Haemoptysis

## Abstract

A case of recurrent haemoptysis due to fibroma is described in a 55 years old male patient. Clinical examination revealed anaemia and bilateral basal crepitations. Chest X - ray showed no abnormality. Bronchoscopy revealed polypoid fibroma in left main bronchus. It was removed bonchoscopically with no recurrence during 12 months follow up.

## INTRODUCTION

Benign turnours of lung comprise fewer than 1 % of all resected lung tumours.^1^ Except hamartomas and carcinoid turnours, all of the benign turnours of lung are rare.^2^ Endobronchial benign tumours often present with features of partial or complete bronchial obstruction, haemoptysis is occasionally noted.^3^ Fibroma of the lung may be pulmonary, endobronchial or pleural in origin. We report a case of endobronchial fibroma presenting with recurrent haemoptysis because of its rarity. The tumour was successfully removed bronchoscopically.

## CASE REPORT

**Fig 1:**
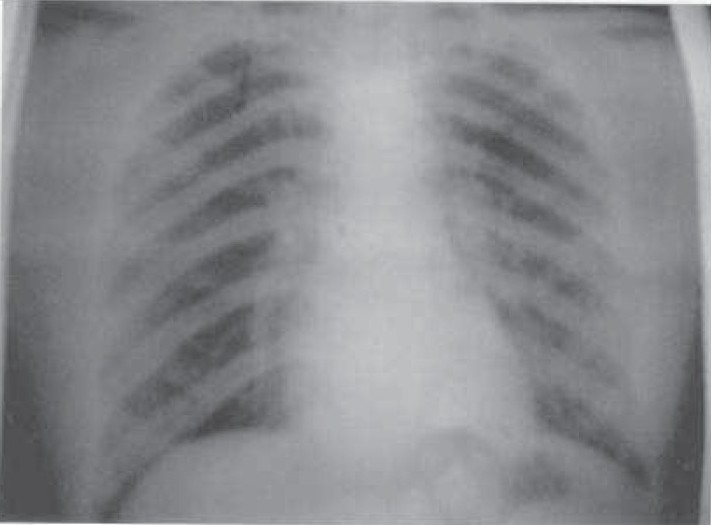
CXR - P A view showing no abnormality.

A 55 year old Hindu male farmer presented with cough, production of white mucoid sputum of moderate amount for one month and recurrent episodes of profuse haemoptysis of same duration. He had no history of fever, dyspnoea, chest pain or bleeding from othet; sites. There was no loss of appetite or weight. There was no history of anti - tuberculous drugs intake in the past. He was normotensive and nondiabetic. He was taking about 10 – 12 bidis / day for last 30 years.

**Fig 2:**
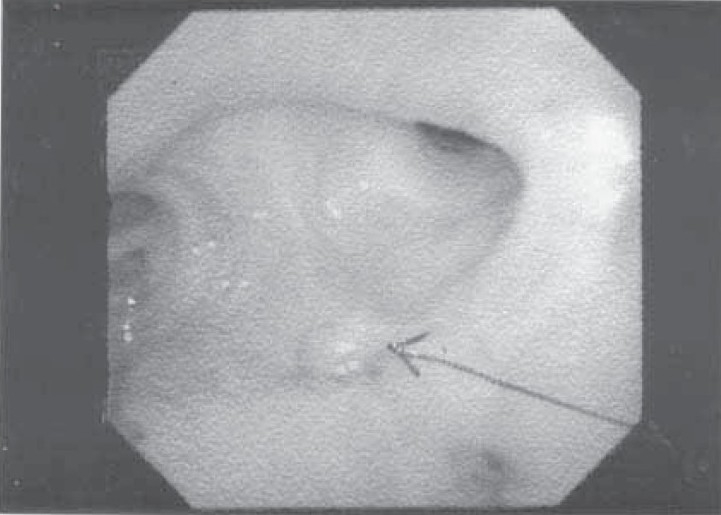
Bronchoscopic view of small polypoid lesion in left main bronchus.

On physical examination, he was of average built and nutrition with moderate anaemia but no clubbing, lymphadenopathy, edema or rise of temperature. Respiratory rate and heart rate were 24 / min. and 12q / min. respectively. His blood pressure was 100 / 76 mm Hg. Examination of respiratory system revealed only bilateral basal coarse crepitations. Examination of other systems was unremarkable.

On admission, he had haemoglobin of 8 g % with normal total and differential white blood cell count and normal platelet count (1.9 Lacs / cmm.). Bleeding time and clotting time were 1 min. 10 sec. and 4 min. 45 sec. respectively. Fasting plasma glucose, serum creatinine, liver function tests were within normal limits. Sputum was negative for acid fast bacilli (AFB). Chest X - ray PA view revealed no abnormality (Fig. 1). HIV 1, 2 serology was non - reactive. The patient was resuscitated with i.v. antibiotics, cough suppressants, hemostatics and 4 units of blood transfusion, following which his general condition improved with rise of haemoglobin to 11.2 g %.

**Fig.3:**
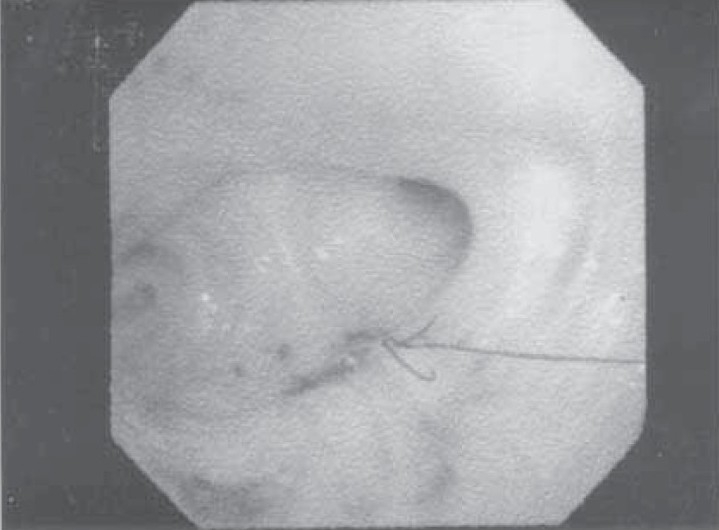
Bronchoscopic view of left main bronchus after removal of polyp.

**Fig.4:**
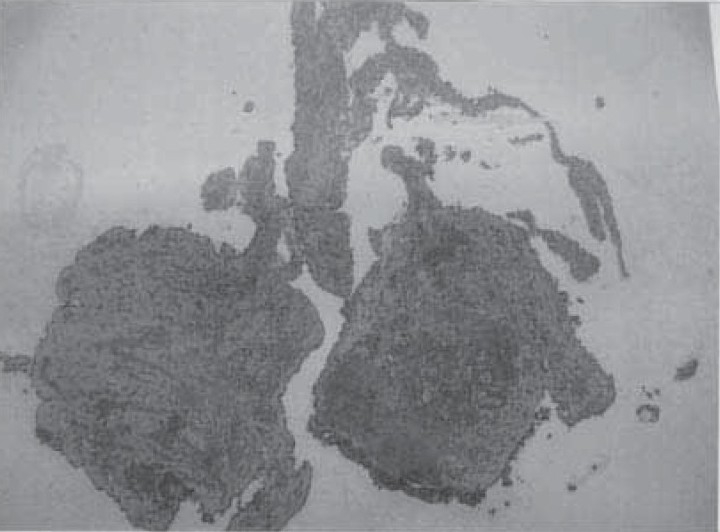
Histopathological appearance of endobronchial fibroma. HIE stain (40× magnification).

Fibreoptic bronchoscopy (FOB) revealed a small polyPOid lesion in the posterior wall of left main bronchus (Fig. 2) and a clot in the apicoposterior segment of left upper lobe bronchus. Bronchial washings and brushings were taken. The polypoid mass was removed bronchoscopically by biopsy forceps (Fig. 3) and sent for histopathological examination. Bronchial washings revealed predominance of neutrophils along with few benign respiratory epithelial cells but no malignant cell and no AFB. Bronchial brushings also showed acute inflammatory cells but malignant cell or AFB was not detected. Histopathological examination of specimen revealed a polyp lined by ciliated pseudostratified columnar epithelium with underlying stroma which was made up of fibrocollagenous tissue, extravasated blood and seromucinous glands, consistent with diagnosis of endobronchial fibroma (Fig.4).

Post - bronchoscopic period was uneventful. During a follow up for 12 months, there was no haemoptysis and follow up bronchoscopy at 12th month revealed no recurrence of the tumour.

## DISCUSSION

Endobronchial benign tumours are frequently symptomatic, often causing partial or complete bronchial obstruction,resultinginrecurrentpneumonia,bronchiectasis, unilateral wheezing, atelectasis, post - obstructive pneumonitis and post obstructive hyperinflation.^3^ Bronchoscopy usually reveals the location of tumours. As the tumours are often covered with normal mucosa, bronchial washings and brushings often non - diagnostic, but forceps biopsy is usually successful in providing a suitable specimen. The lesions are usually completely removable bronchoscopicallr, but sometimes bronchotomy, sleeve dissection or lobectomy may be required.^4^,^5^,^6^

Except hamartomas and carcinoid tumours, all the benign tumours of lung are very rarely reported in literature.^2^ Pulmonary fibroma (also known as fibrous polyp or fibrous tumour) may be parenchymal, endobronchial or pleural. But they are also rarely found in the retroperitoneum, mediastinum, lesser omentum.^7^ Pulmonary parenchymal fibroma is more common in men than in women.^8^ They are usually asymptomatic and detected on routine chest X - ray as a lung mass and diagnosis is obtained by histopathological examination of the resected lung mass.^2^

Pleural fibroma, also known as benign fibrous mesothelioma appears as firm, encapsulated, and lobulated mass with a characteristic whorled appearance.^9^ 70 % of them arise from visceral pleura and the remaining 30% from parietal pleura. They are unrelated to previous asbestos exposure. They appear radiologically as solitary sharply defined, discrete masses at the periphery of the lung.

Endobronchial fibroma generally presents with obstructive pneumonia or atelectasis.^10^ It rarely presents with haemoptysis,^3^ as has occurred in our case. Macroscopically, lesions are well circumscribed with size varying from 1 – 8 cm.^11^ Microscopically, they consist of spindle - shaped fibroblast like cells embedded in a variable amount of collagen. Nuclear atypia is minimal and mitotic figures are sparse or absent. They are readily detachable from bronchial wall during bronchoscopic removal with forceps. They can also be treated with Nd: YAG laser through bronchoscopy or by lobectomy. They generally do not recur, if removal is complete. In our case, the endobronchial fibroma was removed bronchoscopically and there was no recurrence in 12 months follow up.
